# Resveratrol induces PD-L1 expression through snail-driven activation of Wnt pathway in lung cancer cells

**DOI:** 10.1007/s00432-021-03510-z

**Published:** 2021-01-20

**Authors:** Mengmeng Yang, Zongyu Li, Jianping Tao, Hao Hu, Zilin Li, Zhijian Zhang, Feng Cheng, Yihang Sun, Yao Zhang, Jianke Yang, Huijun Wei, Zhihao Wu

**Affiliations:** 1 School of Anesthesiology, Wannan Medical College, Wuhu, 241001 China; 2 Research Laboratory of Tumor Microenvironment, Wannan Medical College, Wuhu, 241001 China; 3 Anhui Province Key Laboratory of Active Biological Macro-Molecules Research, Wannan Medical College, Wuhu, 241001 China; 4 School of Medical Imageology, Wannan Medical College, Wuhu, 241001 China; 5 School of Clinical Medicine, Wannan Medical College, Wuhu, 241001 China; 6 School of Preclinical Medicine, Wannan Medical College, Wuhu, 241001 China

**Keywords:** Resveratrol, PD-L1, Wnt pathway, Snail stability, Axin2

## Abstract

**Purpose:**

Recent clinical trials with agents targeting immune checkpoint pathway have emerged as an important therapeutic approach for a broad range of cancer types. Resveratrol has been shown to possess cancer preventive and therapeutic effects and has potential to be chemotherapeutic agent/adjuvant. Here, we assessed the effect of resveratrol on immune checkpoint pathways.

**Methods:**

The expression patterns of Wnt components and PD-L1 were examined by Western blot, Chromatin immunoprecipitation (ChIP) was used for analysis of DNA–protein interaction, the promoter activity was determined by luciferase reporter assay, apoptosis was analyzed by flow cytometry and the ability of the resveratrol to modulate T cell function was assessed in a co-culture system.

**Results:**

Although the dose-, and cell-type dependent effects of resveratrol on PD-L1 expression have been reported, we show here that resveratrol dose-dependently upregulates PD-L1 expression at the range of pharmacologic-achievable concentrations in lung cancer cells and that is essential for suppression of T-cell-mediated immune response. We also found that Wnt pathway is critical for mediating resveratrol-induced PD-L1 upregulation. Mechanistically, resveratrol activates SirT1 deacetylase to deacetylate and stabilize transcriptional factor Snail. Snail in turn inhibits transcription of Axin2, which leads in disassembly of destruction complex and enhanced binding of β-catenin/TCF to PD-L1 promoter.

**Conclusion:**

We conclude that resveratrol is capable to suppress anti-tumor immunity by controlling mainly PD-L1 expression. This finding will extend the understanding of resveratrol in regulation of tumor immunity and is relevant to the debate on resveratrol supplements for lung cancer patients.

## Introduction

The immunosuppressive ability is an important feature of tumor that enables tumor to grow and metastasize. Human tumors typically exploit immune checkpoint pathway to evade antitumor immunity. Recent clinical trials with agents targeting the programmed cell death 1 (PD-1) and its ligand (PD-L1), which is dominant immune checkpoint pathway operating in tumor microenvironment, has emerged as an important therapeutic approach for a broad range of cancer types. PD-L1, a transmembrane protein found in tumor cell surface, inhibits the immune response upon engagement with its receptor PD-1 expressed on T cells through induction of apoptosis and anergy (a lack of immunologic responsiveness to antigens) in T cells. Thus, upregulation of PD-L1 in cancer cells protects cancers from immune-mediated rejection. Although PD-1/PD-L1 blockade therapy aiming to unleash anti-tumor T cell responses has shown effectiveness and great promise in treating many types of cancers, only a fraction of cancer patients benefits from these therapies. The mechanism that governs the response to anti-PD-L1 therapy remains elusive. Recently, the combination of the anti-PD-L1 with anti-CTLA-4 antibody has been demonstrated to have higher response, but it possesses a cost of significant toxicity and side effects (Allison 2015; Sharma and Allison 2015), highlighting the need to search for the agents suited for combination with immunotherapy.

Because many studies have shown that polyphenols ubiquitously found in plants have immunomodulatory properties (Focaccetti et al. 2019), with the perspective of combining therapies, we sought to identify and characterize the nature of polyphenols that might have potential to influence the immune checkpoint pathways and thus may have therapeutic activities in the combining therapy setting. Resveratrol (3,5,4′-Trihydroxystilbene) is a natural polyphenolic compound found in fruits and vegetables. Studies have revealed a wide spectrum of health benefits of resveratrol, such as anti-oxidant and anti-inflammatory properties. Resveratrol beneficial effects may partially attribute to its ability of modulating a number of molecular targets and pathways, such as the sirtuin 1 (SirT1), a NAD-dependent deacetylase. In the recent past, increasing researches have explored the link between resveratrol and cancer, demonstrating its anti-proliferative, pro-apoptosis and anti-migratory activities (Aluyen et al. 2012; Jang et al. 1997; Singh et al. 2015). Moreover, resveratrol potentiates the efficacy of cancer chemotherapeutic drugs such as cisplatin and doxorubicin in many tumor models and appears to have minimal side effects in human trials (Hsieh and Wu 2010; Patel et al. 2011), holding its great potential as chemotherapeutic agent/adjuvant. Although in vitro and in vivo experimental data are promising for resveratrol’s anti-cancer effects, there are limited researches regarding its involvement in immune checkpoint pathways.

The well-studied mediator of resveratrol’s anti-tumor effect is Wnt signaling pathway (Fu et al. 2014; Singh et al. 2015). Aberrant activation of Wnt cascade has a prominent role in cancer biology. A key step in the regulation of Wnt signaling pathway is the inactivation of the β-catenin destruction complex, which includes scaffold proteins of adenomatous polyposis coli (APC) and Axin2, glycogen synthase kinase 3β (GSK3β) and Casein kinase 1 (CK1) (MacDonald et al. 2009). This complex facilitates phosphorylation and subsequent degradation of β-catenin. Activation of Wnt pathway leads to dissociation of the destruction complex, resulting in β-catenin accumulation and formation of nuclear complex with transcriptional factor TCF/LEF. In this study, we examined the effects of resveratrol on PD-L1 expression and investigated the mechanism underlying the regulation of PD-L1 by resveratrol. We showed that pharmacologic-achievable concentration of resveratrol upregulates PD-L1 expression in lung cancer cells through activation of canonical Wnt signaling pathway and that is essential for suppression of T-cell-mediated immune response. The results are relevant to the debate on resveratrol supplements for lung cancer patients.

## Materials and methods

### Cell culture and transfection

Human lung adenocarcinoma cell lines A549 and H1299 were purchased from National Collection of Authenticated Cell Cultures (Shanghai, China) and cultured with DMEM (Hyclone, Logan, UT, USA) with 10% fetal bovine serum (FBS, Gibco BRL, Grand Island, NY, USA) in a thermostatic incubator containing 5% CO2. Human Jurkat T cell leukemia cells were purchased from National Collection of Authenticated Cell Cultures (Shanghai, China) and were co-cultured with H1299 cells in RPMI-1640 medium (Invitrogen, Burlington, ON, Canada) supplemented with 10% fetal bovine serum for co-culture experiments. A549 and H1299 cells were seeded in a six-well plate until the cell density reached 70–90% confluence, the transfection of plasmids was done with PolyJet DNA Transfection Reagent (SignaGen Laboratories, Gaithersburg, MD, USA) according to the manufacturer’s instructions.

### Small interfering RNA

A549 and H1299 cells seeded in 6-well plates were grown to confluence 30–50% before transfection. Cells were transfected with 10 nM siRNA using 1 μl of GenMute siRNA transfection reagent (SignaGen Laboratories). All the siRNAs were purchased from RiboBio Company Company (Guangzhou, China). The target sequence was as follow: siSirT1, 5′-ACUUUGCUGUAACCCUGUA-3′. After 48 h of transfection, cells were deprived of serum and serum growth factor for 12 h, and then treated with resveratrol for 3 h and harvested.

### Antibodies, reagents and plasmids

Anti-PD-L1 (no.13684), anti-GSK-3β (no.12456), anti-N-cadherin (no.4061), anti-SirT1 (no.8469), anti-β-catenin (no.8480) and anti-Snail (no.3895) were obtained from Cell Signaling Technology (Danvers, MA, USA). Anti-Fibronectin (Ab299) was purchased from Abcam (Cambridge, UK). Anti-β-Actin (A1978), anti-Vimentin (V6630) and anti-FLAG (M2) (F1804) were purchased from Sigma (Sigma, Victoria, BC, Canada). Anti-Ac-lysine (sc-32268) was purchased from Santa Cruz Biotechnology (Santa Cruz, CA, USA). Anti-E-cadherin (13-1700) was obtained from Thermo Fisher Scientific (Waltham, MA, USA). Anti-AXIN2 (A2513) was purchased from ABclonal (ABclonal Cambridge, MA, USA). XAV-939 (HY-15147) was purchased from MedChem Express (MedChem Express, NJ, USA). Resveratrol (R5010) and Cycloheximide (239,763-M) was purchased from SIGMA (Sigma, Victoria, BC, Canada). The Flag-SirT1 (#1781), Flag-SirT1 H363Y (#1792), Snail-pGL2 (#31,694), Flag Snail WT (#16,218) and pcDNA-Wnt3A (#35,908) were purchased from Addgene (Cambridge, MA, USA).

### Cloning and DNA construction

To construct PD-L1 or Axin2 promoters of different lengths, genomic DNA from B2B lung epithelial cells as template was amplified by PCR and the PCR product was cloned into pGL3-BasicVector (Promega, Madison, WI, USA). Point mutations in the PD-L1 or Axin2 promoter were generated by site-specific mutagenesis using the overlap PCR extension method and the longest promoter was used as the template, Axin2 cDNA was amplified using total reverse transcribed cDNA as the template. The amplified PCR fragments were inserted into the pcDNA3. 1 (+) vector. The primers are listed in Table 1.

**Table 1:** Primers used for cloning

Gene	GenBank accession number	Primer (5′–3′)
PD-L1	NM_001314029.1	Fwd: TTGGTACCAGAAGGAAAGGCAAACAACGAAGAGTC Rev: TCTCGAGGGAGCCTCGGGAAGCTGCGCAGAACTG
PD-L1	NM_001314029.1	Fwd: TCTAGAATTAAAACCTTTGCCATATGGGTCT Rev: AGACCCATATGGCAAAGGTTTTAATTCTAGA
PD-L1	NM_001314029.1	Fwd: GACCCTGAGCATTCTCATTTTATGTAGCTCGGGA Rev: TCCCGAGCTACATAAAATGAGAATGCTCAGGGTC
PD-L1	NM_001314029.1	Fwd: ATTTAGAAAA AGAGACATTTTAGAAAAGGGAGCA Rev: TGCTCCCTTTTCTAAAATGTCTCTTTTTCTAAAT
AXIN2	NM_004655.3	Fwd: CCCAAGCTTATGAGTAGCGCTATGTTGGT Rev: CGGGGTACCTCAATCGATCCGCTCCACTT
AXIN2	NM_004655.3	Fwd: CGGGGTACCGGCGCCGGGCCTTTCTTTATGTT Rev: CATGCTAGCGAGTGAGTTGGTTTCTGGTTTGGG
AXIN2	NM_004655.3	Fwd: CCGCTAGGCTGTGTGAGTCACAAACCAATTTCGGTA Rev: TACCGAAATTGGTTTGTGACTCACACAGCCTAGCGG

### Western bolt analysis

The protein was extracted from the cells with the lysis solution Sample Buffer, (Laemmli 2 × Concentrate, S3401; SIGMA), and transferred to the nitrocellulose (NC) membrane (GE Healthcare, Piscataway, NJ, USA) by polyacrylamide gel electrophoresis, then blocked with 5% skim milk for 1 h at room temperature. The signals were detected by a chemiluminescence detector (Amersham Imager 600 System) by incubation with various primary antibodies and corresponding secondary antibodies.

### Quantitative real-time RT–PCR analysis

Total mRNA was isolated from cells using TRIzol according to the manufacturer's protocols. Total RNA obtained was reversely transcribed into cDNA by PrimeScript First Strand cDNA Synthesis Kit (TaKaRa Bio, DaLian, China) using ABI SYBR Green Master Mix (Applied Biosystems, Carlsbad, CA, USA), The mixture was amplified by the ABI 7500 Real-time PCR System (Applied Biosystems). Each sample was repeated in triplicate and analyzed using the Quantification Software (Applied　Biosystems). All RT-PCR Primer sequences are shown in Table 2.

**Table 2:** Primers used for RT‐PCR

Gene	GenBank accession number	Primer (5′–3′)
PD-L1	NM_001314029.1	Fwd: ACTGGCATTTGCTGAACGC Rev: ACAATTAGTGCAGCCAGGTCT
GAPDH	NM_002046	Fwd: GACCCCTTCATTGACCTCAAC Rev: CTTCTCCATGGTGGTGAAGA
Axin2	NM_004655.3	Fwd: CAGGACACTGCTCTCTCAGATTCA Rev: TCACAACAGCCTTTGCAGGG-
Snail	NM_005985.4	Fwd: AGGCAGCTATTTCAGCCTCC Rev: CACATCGGTCAGACCAGGC
β-actin	NM_001101.5	Fwd: CCTTCCTGGGCATGGAGTCCT Rev: GGAGCAATGATCTTGATCTTC

### Dual luciferase reporter assays

The reporter gene plasmid was transfected into the cells. After 48 h, the cells were collected with a PLB lysate into a 1.5 ml centrifuge tube and subjected to high speed centrifugation, and the activities of firefly luciferease and Renilla luciferase were analyzed by dual luciferase reporter assay following the manufacturer's protocol (Promega, Madison, WI, USA). Each experiment was repeated in triplicate using a multimode microplate reader (TriStar LB941, Berthold Technologies, Bad Wildbad, Germany). The results are expressed as a mean of triplicates ± SD.

### Flow cytometric assessment of apoptosis

H1299 cells were first treated with 20 μM resveratrol in RPMI-1640 medium for 3 h, the medium then was removed and resveratrol-treated cells immediately were co-cultured with Jurkat-T cells in RPMI-1640 medium, apoptosis was detected using Annexin V-FITC Apoptosis Detection Kit (KeyGEN Biotech, Jiangsu, China). Full flow cytometry analysis was performed on a BD Facs Verse Flow Cytometer (Becton Dickinson, Franklin L, NJ, USA). The apoptotic index was determined by the percentage of apoptotic Jurkat T cells. Three independent experiments were performed.

### Co-immunoprecipitation (Co-IP) assays

To investigate the interaction between SirT1 and Snail, A549 and H1299 cells were grown to 80–90% confluence. Cells were washed with ice-cold PBS three times before lysis in IP lysis buffer. After removing the insoluble matter by centrifugation at 12,000×*g*, the protein was assayed using the Bradford concentration method (Bio-Rad, Hercules, CA, USA). Pre-cleared lysates with equal amounts of protein were incubated with anti-Snail, anti-SirTl and anti-Acetyl-lysine antibodies overnight at 4 °C. Protein G-agarose beads (P3296, SIGMA) were then added to the immunoprecipitation (IP) mixture for 2 h. The beads were collected and buffered three times with lysate. The precipitated protein was eluted and denatured in 2 × SDS loading buffer analyzed by western blotting analysis.

### Chromatin immunoprecipitation (ChIP) assay

ChIP assay was performed using the SimpleChIP Enzymatic Chromatin IP Kit (Cell Signaling Technology). Cells were washed with ice-cold PBS and then were lysed with the lysis buffer provided in the kit. Chromatin preparation and immunoprecipitation were performed according to the manufacturer's instructions. Chromatin was immunoprecipitated using anti-β-catenin or anti-FLAG (M2) antibody. Normal rabbit IgG (Cell Signaling Technology) was used as a negative control. The immunoprecipitated DNA and the input DNA were extracted by reversing the cross-linking. PCR and RT-PCR were performed using purified DNA as a template. The PCR products were electrophoresed on a 1% agarose gel. The qRT-PCR results were analyzed according to the protocols. All CHIP Primer sequences are shown in Table 3.

**Table 3:** Primers used for Chromatin immunoprecipitation

Gene	GenBank accession number	Target sequence (5′–3′)
PD-L1	NM_001314029.1	Fwd: GGCAAACAACGAAGAGTCCAATTTCT Rev: TATCCCGCGCTGAACTTCTAGGTG
Axin2	NM_004655.3	Fwd: GACACAACCTTCCAAAAACCCA Rev: AGGGCCGAGTGGGAAATAT

### Data availability

To analyze the correlation between Snail and PD-L1, we performed data mining on the GEO database (https://www.ncbi.nlm.nih.gov/gds/). GSE33532 was based on the GPL570 platform (HG-U133_Plus_2, Affymetrix Human Genome U133 Plus 2.0 Array), and including 80 non-small lung cancer and 20 normal tissues. The gene correlations were analyzed using the Cancer Genome Atlas (TCGA) data (RNA-SeqHTSeq-FPKMUQ) in lung adenocarcinoma (*n* = 534) (http://tcga-data.nci.nih.gov). RNA-seq number values were matched with the gene expression. The Spearman’s rank correlation coefficient (rho) between SNAI1 gene expression and Axin2 was calculated. Similarly, we analyzed the correlation between Snail and PD-L1 in GSE33532. Statistical analyses and data generation were carried out using GraphPad Prism. (GraphPad, San Diego, CA, USA).

### Statistical analysis

In general, unpaired two-tailed Student t-test and one-way analysis of variance (ANOVA) were used to make inter-group comparison. All statistical analysis was performed using SPSS 13.0 Statistical Software (SPSS Inc, Chicago, IL, USA). All results were presented as a mean ± s.d. (standard deviation) with a *P*-value < 0.05 considered statistically significant.

## Results

### Resveratrol activates Wnt pathway to upregulate PD-L1 expression

**Fig. 1:**
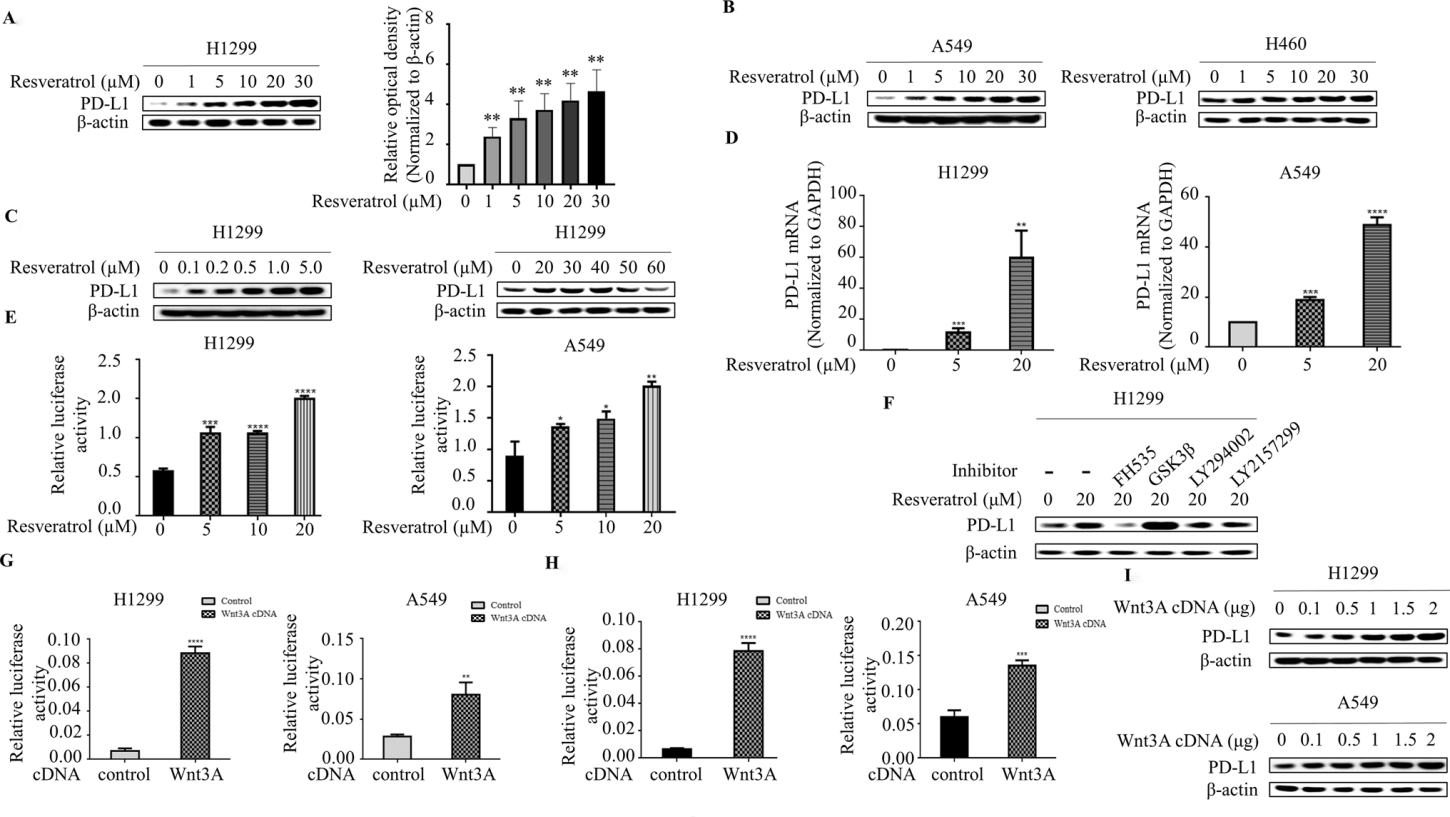
Resveratrol activates Wnt pathway to upregulate PD-L1 expression. **a** Western blot demonstrates increased PD-L1 expression following 3 h of resveratrol (0, 1, 5, 10, 20 and 30 μM) stimulation in lung cancer cell lines H1299 (left panel) and the levels of PD-L1 was quantified by densitometry (right panel) (**p < 0.01, for difference from untreated control cells by ANOVA with Dunnett’s correction for multiple comparisons). **b** Resveratrol also increased PD-L1 expression in A549 and H460 cells. **c** Western blot demonstrates increased PD-L1 expression following 3 h of low-dose resveratrol (< 5 μM) stimulation but decreased PD-L1 expression following 3 h of high-dose resveratrol (> 40 μM) stimulation in H1299 cell line. **d** RNA was extracted and subjected to qRT-PCR in A549 and H1299 cells with indicated resveratrol treatment. Values represent the relative reduction of PD-L1 mRNA levels normalized to GAPDH. The bars represent the mean ± SD of triplicates (**p < 0.01, ***p < 0.001, ****p < 0.0001 for difference from untreated control cells by ANOVA with Dunnett’s correction for multiple comparisons). **e** A549 and H1299 cells were co-transfected with PD-L1 promoter and control Renilla luciferase reporter gene plasmid and treated with indicated resveratrol concentrations after 48 h. Luciferase activity was determined and normalized using the dual luciferase reporter system (*p < 0.05, **p < 0.01, ***p < 0.001, ****p < 0.001 for the difference from the control cells by ANOVA with Dunnett’s correction for multiple comparisons). **f** Representative Western blot analysis of PD-L1 in H1299 cells pre-treated with FH535, GSK3β, LY294002, LY2157299 at a concentration of 2 μM for 1 h and then with Resveratrol for 3 h. **g** A549 and H1299 cells were transiently co-transfected with Wnt3A cDNA, along with the TOPflash luciferase reporter, and luciferase activity was determined after transfection for 48 h and normalized using the dual luciferase reporter system. The bars represent the mean ±sSD of triplicates (**p < 0.01, ****p < 0.0001 for difference from untreated control cells by ANOVA with Dunnett’s correction for multiple comparisons). **h** A549 and H1299 cells were transiently co-transfected with Wnt3A cDNA, along with the PD-L1 promoter reporter construct, and luciferase activity was determined and normalized after transfection for 48 h using the dual luciferase reporter system (***p < 0.001, ****p < 0.001 for the difference from the control cells by ANOVA with Dunnett’s correction for multiple comparisons). **i** Overexpression of Wnt3A increased PD-L1 expression in dose-dependent manner

Resveratrol was shown to function as an immunomodulatory molecule and affect tumor immunotherapy (Focaccetti et al. 2019). It is unknown whether resveratrol plays a role in immune checkpoint pathway in lung cancer cells. To dress this question, we evaluated whether resveratrol affects the PD-L1 expression in human lung cancer cells. The cells were starved and subsequently treated with various concentrations of resveratrol for 3 h. The concentration of resveratrol exploited in this study is within the range of dose recommendation for dietary resveratrol (Castillo-Pichardo et al. 2013), which is relevant for a better understanding of the effect of resveratrol. In stark contrast with our prediction of anti-cancer effect of resveratrol, resveratrol treatment triggered a robust induction of PD-L1 expression in a dose-dependent manner in H1299 cells (Fig. 1a). The ability of resveratrol to induce PD-L1 expression is not limited to H1299 cells, similar results were observed in A549 as well as H460 lung carcinoma cells (Fig. 1b). Accumulating evidence indicates that resveratrol effects are concentration-dependent (Focaccetti et al. 2019; Xu et al. 2017). Therefore, we analyzed the PD-L1 expression from the cells treated with relatively low- and high-dosages of resveratrol. In H1299 cells, PD-L1 expression was still induced following the exposure to a low dosage (below 5 μM) of resveratrol (Fig. 1c); however, at a higher dose (> 40 μM), resveratrol treatment resulted in a progressive reduction of PD-L1, indicating that PD-L1 expression can be regulated by resveratrol in a dose-dependent manner.

It has been reported previously that PD-L1 expression is sensitive to regulation at both the transcriptional and post-transcriptional levels (Wang et al. 2018). Therefore, we sought to determine how PD-L1 expression is regulated by resveratrol. We first measured the mRNA levels of PD-L1 by quantitative real-time PCR analysis. We found that resveratrol dose-dependent increased PD-L1 mRNA levels in both H1299 and A549 cells (Fig. 1d). To further investigated whether resveratrol is able to control the activity of the PD-L1 promoter, the cells were transfected with a luciferase reporter plasmid containing human PD-L1 promoter. Treatment of resveratrol resulted in an increase of the PD-L1 promoter-driven luciferase activity compared with control (Fig. 1e), indicating that resveratrol transcriptionally regulates PD-L1 expression.

To interrogate the mechanisms by which resveratrol regulates PD-L1 expression, we utilized small molecule kinases inhibitors to determine whether Wnt, Akt and TGF-β, signaling pathways known to be regulated by resveratrol, is involved in resveratrol-induced PD-L1 expression. In contrast to Akt inhibitor LY294002 and TGF- β inhibitor LY2157299, which had no effect, Wnt inhibitor FH535 caused substantial reduction of PD-L1 expression in the presence of resveratrol (Fig. 1f). In line with the proposition that GSK-3 β negatively regulates canonical Wnt pathway, the ability of resveratrol to increase PD-L1 protein levels was largely enhanced by addition of GSK-3 β inhibitor, implicating that Wnt pathway may contribute to resveratrol-induced PD-L1 expression. To test the possibility that Wnt might regulate PD-L1 expression, cells were transfected with Wnt3A cDNA. As shown in Fig. 1g, overexpression of Wnt3A increased the transcriptional activity of Wnt3A-dependent reporter TOPflash, indicating that the downstream components of Wnt pathway were intact. Strikingly, the transcriptional activity of PD-L1 promoter reporter was markedly induced by Wnt3A overexpression (Fig. 1h). Furthermore, overexpression of Wnt3A markedly increased PD-L1 protein levels (Fig. 1i). Those findings reinforce the notion that Wnt pathway is functional relevant to resveratrol-induced PD-L1 expression.

### β-catenin/TCF transcriptional complex is required for resveratrol-induced PD-L1 expression

**Fig. 2:**
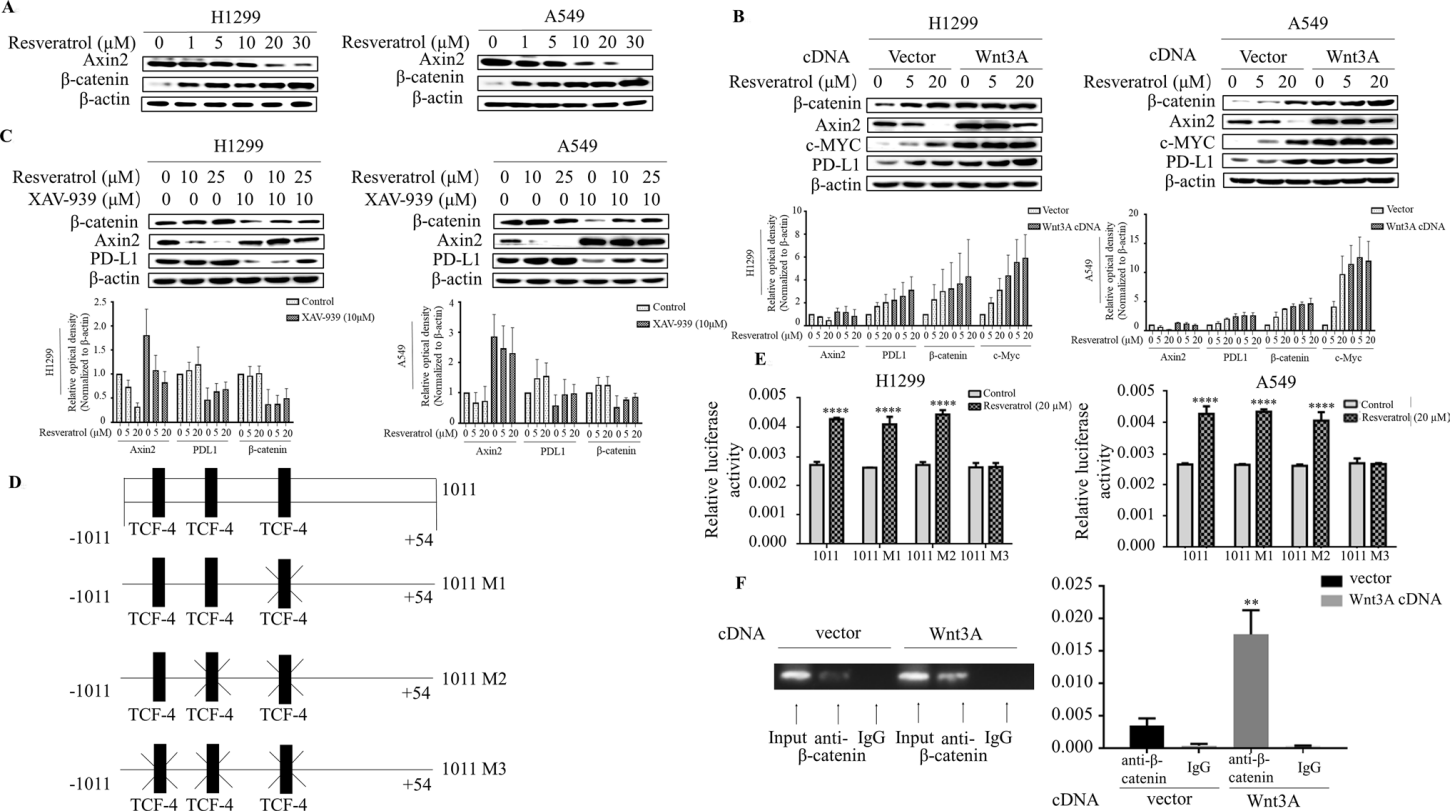
β-catenin/TCF transcriptional complex is required for resveratrol-induced PD-L1 expression. **a** Western blot analysis of Axin2 and β-catenin in H1299 and A549 cells treated with indicated amount of resveratrol, and it showed that Wnt pathway is activated by resveratrol. **b** Western blot analysis of Axin2, β-catenin, and PD-L1 in indicated cells at day 3 after transfection with Wnt3A cDNA or empty vector and then treated with resveratrol (upper panel) and the quantification of each protein levels by densitometry were shown in lower panel. **c** Western blot analysis of Axin2, β-catenin and PD-L1 in A549 and H1299 cells treated with XAV-939 at a concentration of 10 μM for 1 h and then with different concentrations resveratrol for 3 h (upper panel) and quantification was in lower panel. **d** Scheme of PD-L1 luciferase reporter constructs containing three potential TCF-4 binding sites (1011) with different mutations (1011 M, 1011 M2 and 1011 M3). Mutations were made in sequence at three potential TCF-4 binding sites (1011 M1: CCAAAG to CCTTTG, 1011 M2: TAAAAG to CATTTT on the construct of 1011 M1 and 1011 M3: AAAAAA to CATTTT on the construct of 1011 M2). **e** The cells were transiently transfected with different PD-L1 promoter reporters for 48 h and treated with resveratrol for 3 h. Luciferase activity was determined and normalized using the dual luciferase reporter system (****p < 0.001 for the difference from the untreated reporter-transfected cells by ANOVA with Dunnett’s correction for multiple comparisons). **f** ChIP assays were performed using anti-β-catenin antibodies. The standard PCR product is run and scan (left panel). Quantitative RT-PCR results were expressed using histograms (right panel). The bars represent the mean ± SD of triplicates (**p < 0.01 for difference from empty control plasmid-transfected cells by ANOVA for multiple comparison)

A key step in the induction of Wnt pathway is inactivation of the β-catenin destruction complex, which includes GSK-3β, Axin2 and APC. We sought to determine whether resveratrol regulates PD-L1 through the same pathway. For this, we performed Western blot to examine resveratrol-dependence of those gene expression in lung cancer cells. β-catenin levels were dramatically induced by resveratrol (Fig. 2a). Consistent with previous studies, Wnt3A increased Axin2 expression (compare lane 1 and lane 4) (Fig. 2b), interestingly, resveratrol decreased Axin2 levels in a dose-dependent manner in the presence and absence of Wnt3A. Since Axin2 functions as scaffolding protein for the β-catenin destruction complex, we thus considered the possibility that resveratrol-induced decreases in Axin2 levels may affect PD-L1 protein levels. Indeed, stabilization of β-catenin destruction complex in resveratrol-treated cells using XAV939, a small molecule that promotes Axin stabilization, diminished effect of resveratrol on PD-L1 induction (Fig. 2c). Since Wnt destabilizes β-catenin destruction complex independent of Axin levels, resveratrol-induced PD-L1 levels could conceivably be increased further in cells transfected with Wnt3A. Indeed, resveratrol appeared to synergize with Wnt3A action, increased PD-L1 levels by resveratrol were massively augmented in the cells transfected with Wnt3A (Fig. 2b), indicating that destabilization of β -catenin destruction complex is crucial in regulation of PD-L1 by resveratrol.

Finding that the β-catenin destruction complex regulates PD-L1 hints the requirement of β-catenin for induction of PD-L1 levels. When destabilization of β-catenin destruction complex occurs, β-catenin is stabilized in the cytoplasm, and subsequently translocated into nucleus to complex with TCF/LEF to activate the gene transcription. Examination of the sequence of the region of upstream of transcriptional start site in the PD-L1 gene showed three putative TCF-4 binding sites (Fig. 2d). To determine whether β-catenin/TCF is able to mediate resveratrol-induced PD-L1 expression, three putative TCF-4 binding sites in the PD-L1 promoter reporter construct were individually mutated and the luciferase activity was monitored in the cells treated with resveratrol. The construct contains all three predicted TCF-4 binding sites (1011) showed a significant induction of luciferase activity by resveratrol treatment (Fig. 2e). Interestingly, constructs containing mutations of either the one site (1011 M1) or the two sites (1011 M2) relative to transcriptional start site still had resveratrol-induced promoter activity as significantly as wild-type construct (1011). Remarkably, the responsiveness of promoter activity to resveratrol diminished when the third TCF-4 binding site was mutated. Furthermore, we carried out the chromatin immunoprecipitation (ChIP) assay to immunoprecipitate the region covering the third TCF-4 binding site (Fig. 2f), ChIP with anti- β-catenin confirmed that enhanced binding of b-catenin/TCF4 to PD-L1 promoter in H1299 cells transfected with Wnt3A. Taken together, these results indicated the canonical Wnt pathway mediated resveratrol-induced PD-L1 expression.

### Resveratrol induces Snail-dependent reduction of Axin2 levels

**Fig. 3:**
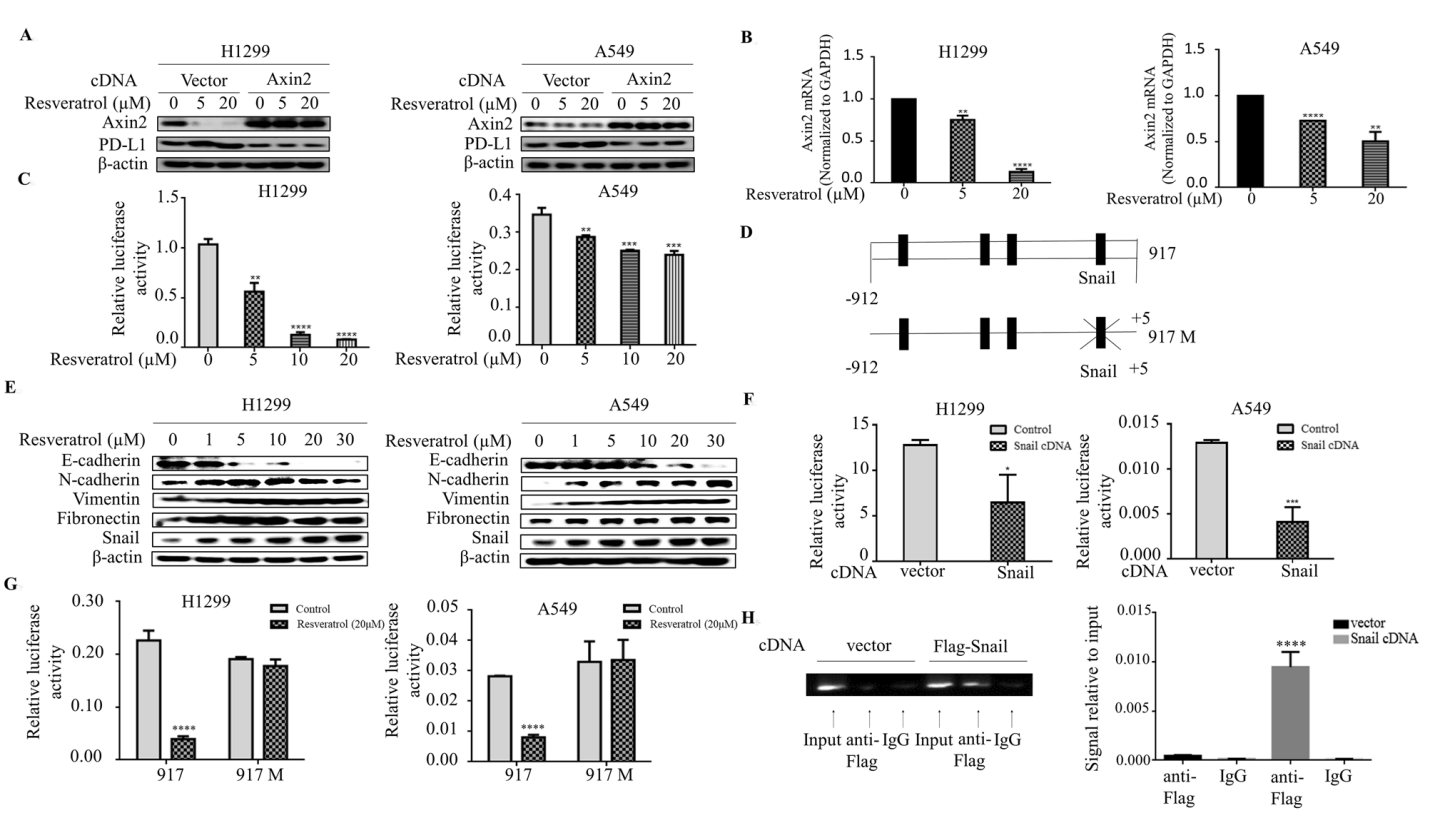
Resveratrol induces Snail-dependent reduction of Axin2 levels. **a** Western blot analysis of Axin2 and PD-L1 in H1299 and A549 cells at day 3 after transfection with Axin2 cDNA or vector and then treated with resveratrol for 3 h. **b** RNA was extracted and subjected to qRT-PCR in A549 and H1299 cells with indicated concentrations of resveratrol treatment. Values represent the relative reduction of Axin2 mRNA levels normalized to GAPDH. The bars represent the mean ± SD of triplicates (**p < 0.01, ****p < 0.0001 for difference from control cells by ANOVA with Dunnett’s correction for multiple comparisons). **c** A549 and H1299 cells were co-transfected with Axin2 promoter reporter construct and control Renilla luciferase reporter gene plasmid and treated with indicated resveratrol concentrations after 48 h. Luciferase activity was determined and normalized using the dual luciferase reporter system (**p < 0.01, ***p < 0.001, ****p < 0.001 for difference from control cells by ANOVA with Dunnett’s correction for multiple comparisons). **d** Schematic representation of potential Snail binding sites in the Axin2 promoter. The mutation construct (917M) was done from CAGGTG to AGTCAC at the potential Snail binding site. **e** Western blot examines epithelial marker E-cadherin, mesenchymal markers N-cadherin, Fibronectin, Vimentin, and EMT inducer Snail in H1299 and A549 cells with different doses of resveratrol treatment. **f** A549 and H1299 cells were transiently co-transfected with Snail cDNA, along with the Axin2 promoter reporter, and luciferase activity was determined and normalized after transfection for 48 h using the dual luciferase reporter system (*p < 0.05, ***p < 0.001, for difference from vector-transfected cells by ANOVA with Dunnett’s correction for multiple comparisons). **g** The cells were transiently transfected with wild-type and mutated Axin2 promoter construct for 48 h and treated with resveratrol for 3 h. Luciferase activity was determined and normalized using the dual luciferase reporter system (****p < 0.001 for difference from untreated transfected-cells by ANOVA with Dunnett’s correction for multiple comparisons). **h** ChIP assays were performed using anti-FLAG antibodies. The standard PCR product is run and scan (left panel). Quantitative RT-PCR results were expressed using histograms (right panel). The bars represent the mean ± SD of triplicates (****p < 0.0001 for difference from empty control plasmid-transfected cells by ANOVA for multiple comparison)

To ascertain whether resveratrol acts through Axin2 to induce PD-L1 expression, we first utilized overexpression approaches to determine whether Axin2 is sufficient to suppress resveratrol-induced PD-L1 levels. Indeed, introduction of Axin2 into both H1299 and A549 cells is sufficient to inhibit the PD-L1 levels by resveratrol (Fig. 3a). Next, we sought to determine by which mechanism resveratrol suppresses Axin2 expression. qRT-PCR analysis revealed that mRNA levels of Axin2 decreased in parallel with its protein levels after resveratrol stimulation in both cell lines (Fig. 3b). Moreover, we used dual luciferase reporter assay to analyze the effect of resveratrol on the Axin2 promoter activity. Markedly, the promoter activity of Axin2 was significantly suppressed by resveratrol in both H1299 and A549 cells, indicating that resveratrol also regulates transcription of Axin2 (Fig. 3c). Analysis the genomic sequence upstream of the start codon of Axin2 showed four putative Snail binding sites (Fig. 3d). Snail, a transcriptional repressor, is a potent inducer of epithelial-mesenchymal transition (EMT). Since the canonical Wnt signaling also engages neoplastic EMT program and Snail expression, we asked whether Snail might also play an important role in Axin2 regulation. Stimulation of resveratrol dose-dependently increased Snail levels in association with the suppression of E-cadherin protein levels, as well as induction of N-cadherin, Fibronectin and Vimentin levels (Fig. 3e), consistent with the notion that Snail might be relevant to this process. Importantly, the promoter activity of Axin2 was suppressed significantly by Snail overexpression (Fig. 3f). Interestingly, the responsiveness of the Axin2 promoter activity to Snail overexpression disappeared when the putative Snail binding site nearest to transcription start site was mutated (Fig. 3g), indicating an essential role of this Snail binding site in Axin2 regulation. Furthermore, ChIP assay was performed in the lysates prepared from cells transfected either with vector or Flag-Snail cDNA. ChIP analyses confirmed that anti-Flag antibodies specially immunoprecipitated the DNA fragment containing this Snail binding site (Fig. 3h). Taken together, these results suggested that resveratrol promotes Snail-dependent reduction of Axin2 levels.

### Resveratrol-activated SirT1 deacetylates Snail and increases Snail stability

**Fig. 4:**
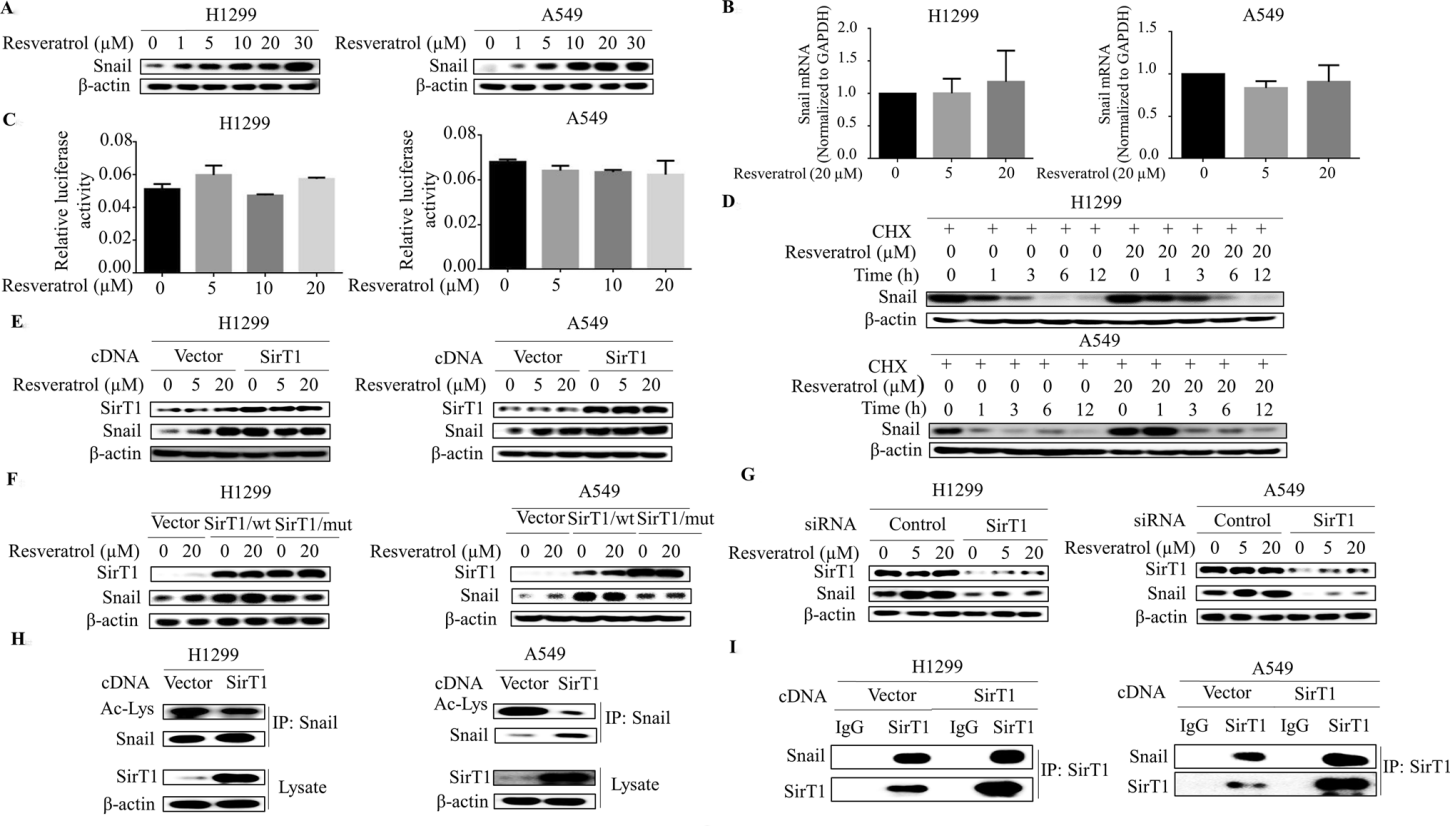
Resveratrol-activated SirT1 deacetylates Snail and increases Snail stability. **a** Western blot demonstrates increased Snail expression following 3 h of resveratrol (0, 1, 5, 10, 20 and 30 μM) stimulation in different histological non-small-cell lung cancer cell lines. **b** RNA was extracted and subjected to qRT-PCR in A549 and H1299 cells with indicated resveratrol treatment. Values represent the relative reduction of Snail mRNA levels normalized to GAPDH. No significant differences were observed (difference from untreated cells by ANOVA with Dunnett’s correction for multiple comparisons was calculated). **c** A549 and H1299 cells were co-transfected with Snail promoter reporter and control Renilla luciferase reporter gene plasmid for 48 h and then treated with resveratrol for 3 h. Luciferase activity was determined and normalized using the dual luciferase reporter system. No significant differences were found. **d** The half-life of Snail was increased in cells treated with resveratrol compared with that in control cells, as demonstrated by CHX chase assays. Cells were exposed to CHX and collected at the indicated times. Cell lysates were immunoblotted with the indicated antibodies. **e** Western blot analysis of Snail in H1299 and A549 cells at day 3 after transfection with SirT1 cDNA or control vector and with resveratrol treatment. **f** Western blot analysis of Snail in the cells with resveratrol treatment at day 3 after transfection with wild-type SirT1 cDNA (SirT1/wt) or acetylase-dead SirT1 construct (SirT1/Mut) or empty vector. **g** Western blot analysis of Snail in the cells at day 3 after transfection with SirT1 siRNA or control siRNA. **h** Cells were transfected with SirT1 for 48 h, and then cell homogenates were immunoprecipitated with anti-Snail and blotted with anti-acetyl-lysine. **i** Co-immunoprecipitation assays were used to detect the direct interaction of SirT1 and Snail after transfection with SirT1 in the H1299 and A549 cells

We next dissected the molecular mechanism by which resveratrol regulates Snail expression in lung cancer cells. Western blot demonstrated that resveratrol dose-dependently increased the levels of Snail protein (Fig. 4a), we then questioned whether resveratrol transcriptionally activates Snail expression. To examine this, mRNA level of Snail was analyzed by qRT-PCR, unexpectedly, Snail mRNA levels was not affected by resveratrol treatment (Fig. 4b). In tandem fashion, resveratrol did not cause discernable change in the Snail promoter activity (Fig. 4c), suggesting that Snail is regulated post-transcriptionally by resveratrol. To test this possibility, we examined Snail stability by CHX chase experiments. As shown in Fig. 4d, Snail is relatively unstable protein; however, Snail half-life is increased substantially by resveratrol. Hence, resveratrol decreased Axin2 expression by allowing for increased Snail stability.

The stability of Snail protein can be regulated post-transcriptionally by phosphorylation and acetylation. SirT1, a NAD^+^-dependent deacetylase and major target of resveratrol, is also involved in tumorigenesis. This raises the possibility that SirT1 might control Snail acetylation levels, and hence Snail protein stability. To evaluate this model directly, cells were transfected with SirT1 expression vector and Snail protein levels were assessed. As shown in Fig. 4e, the steady-state concentrations of Snail protein were increased dramatically in the SirT1-transfected cells. Strikingly, a catalytically inactive SirT1 mutant had no effect on Snail levels but sufficient to impair Snail induction by resveratrol (Fig. 4f), indicating SirT1 is an essential mediator of resveratrol-induced Snail levels in lung cancer cells. Likewise, when cells were transfected with SirT1 siRNA, resveratrol-induced Snail levels were predictably repressed (Fig. 4g). Moreover, immunoprecipitation of homogenates from SirT1-transfected cells with anti-Snail followed by western blot with anti-acetylated-lysine showed that SirT1 specifically decreased acetylation levels of Snail protein (Fig. 4h), and co-immunoprecipitation also confirmed that Snail interacted with SirT1 in SirT1-tranfected cells (Fig. 4i). Taken together, these data suggest that resveratrol-SirT1 pathway increases Snail stability by decreasing its acetylation.

### Resveratrol suppresses the proliferation and cytokine secretion by T cells

**Fig. 5:**
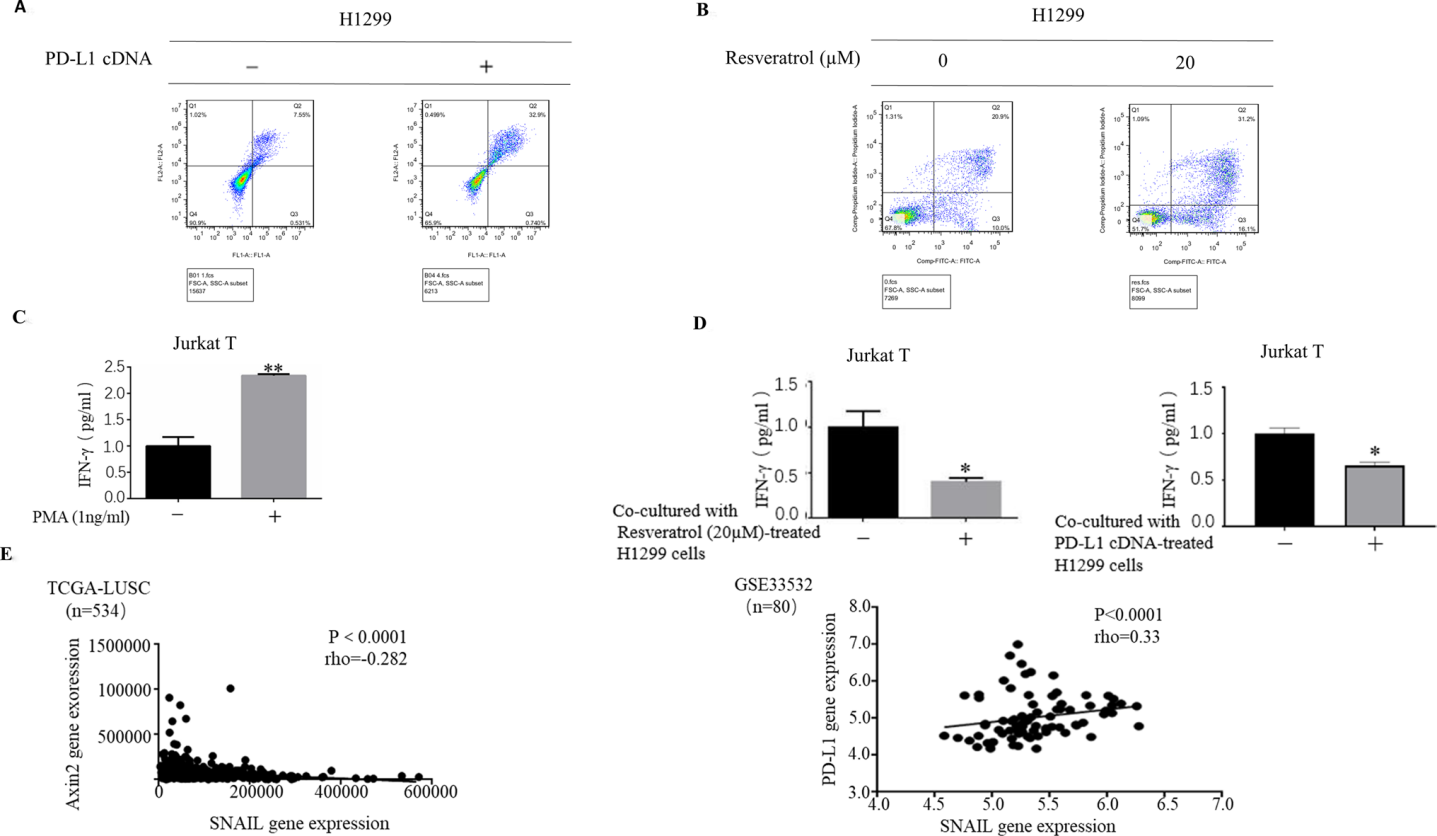
Resveratrol suppresses the proliferation and cytokine secretion by T cells. **a**, **b** Flow cytometric assessment of apoptosis showed that apoptosis levels of Jurkat T cells co-cultured either with PD-L1 overexpressed H1299 cells (**a**) or resveratrol treated H1299 cells (**b**) are increased. **c** Jurkat T cells were grown onto six-well plates and then treated with PMA (100 ng/ml) for 12 h. IFN-γ levels is strongly increased in Jurkat T cells stimulated with PMA. **d** PMA-stimulated Jurkat T cells were co-cultured either with resveratrol-treated or PD-L1 overexpressed H1299 cells. Compared with cells co-cultured with resveratrol-untreated and PD-L1 cDNA untransfected H1299 cells, resveratrol-treated and PD-L1 overexpressed H1299 cells inhibit IFN-γ production of co-cultured Jurkat T cells. **e** The correlations between Snail and PD-L1 expression from GEO database and the correlations between Snail and Axin2 expression from TCGA data set (TCGA-LUSC). The Spearman’s rank correlation coefficient ($\rho$) and the *p* values were calculated

In neoplastic state, upregulation of PD-L1 are known as triggering the anergy and/or apoptosis of the T cells. As such, the ability of the resveratrol to modulate T cell function was assessed in a standard co-culture system. We used Jurkat T leukemia cells, which not only have a characteristic features of T lymphocytes but also express high levels of PD-1, in our co-culture experiments. We first examined apoptosis (annexin V labeling) in Jurkat T cells co-cultured with PD-L1 overexpressed H1299 cells by flow cytometry. As expected, PD-L1 overexpression caused a robust increase in apoptosis of Jurkat T cell (Fig. 5a). Significantly, Jurkat T cells co-cultured with resveratrol-treated cells also showed remarkable induction of apoptosis (Fig. 5b).

We next addressed the role of resveratrol in production of cytokines such as interferon-γ (IFN-γ) in Jurkat T cells in our co-culture experiments. We first investigated whether phorbol 12-myristate 13-acetate (PMA) could serve as an external stimulus for increasing production of IFN-γ in Jurkat T cells. Indeed, Jurkat T cells produced significant amount of IFN-γ upon stimulation of PMA (Fig. 5c). Remarkably, co-culture of PMA-stimulated Jurkat T cells either with resveratrol-treated cells or with PD-L1 overexpressed H1299 cells was sufficient to impair IFN-γ production by Jurkat T cells (Fig. 5d). Overall, these data indicated the role of resveratrol in suppression T cell function.

Finally, we performed further analysis to query the publicly available database from the Cancer Genome Atlas (TCGA) and Gene Expression Omnibus (GEO, http://www.ncbi.nlm.nih.gov/geo/). Consistent with our results that Snail plays critical role in mediating resveratrol-stimulated PD-L1 expression by reducing Axin2 levels, we found that the expression levels of Snail inversely correlated with Axin2 levels (TCGA-LUSC, *R* = − 0.282, p < 0.0001) and positively correlated with PD-L1 levels (GSE33532, *R* = − 0.33, *p* < 0.0001) (Fig. 5e), indicating the relevance of our findings in human malignancy.

## Discussion

Immune checkpoint blockade therapy has revolutionized cancer treatment and improved greatly the outcome of several types of cancer including non-small cell lung cancer. However, only a subset of patients with such a treatment displayed a strong response. Therefore, the immunomodulatory strategies for eliciting and enhancing anti-tumor immunity have been developed and currently focus on combining therapies (Melero et al. 2015). Resveratrol is an extensively studied polyphenols with remarkable immunomodulatory ability in regulating human immune system (Yahfoufi et al. 2018), most published studies have presented anti-tumor property of resveratrol (Aluyen et al. 2012; Singh et al. 2015). Recently, several studies provided evidences showing that the levels of PD-L1 was significantly increased in the breast and colon cancer cells treated with resveratrol at supra-pharmacological concentrations (> 50 μM) and, in contrast, resveratrol antagonizes thyroid hormone-induced PD-L1 expression in oral cancer cells also at relatively high concentrations (> 40 μM) (Lin et al. 2019; Lucas et al. 2018), indicating that effects of resveratrol on PD-L1 expression could be the dose-, and cell-type dependent. However, none of the studies have examined the role of resveratrol in PD-L1 expression in lung cancers at pharmacologic-achievable concentration. In this study, we used two NSCLC cell lines to systematically address the role of resveratrol in PD-L1 expression. Unexpectedly, we demonstrated that resveratrol dose-dependently increased PD-L1 expression in NSCLC cells and the resveratrol treatment in our co-culture model strongly suppressed T cell function. We also found that Wnt pathway is critical for mediating resveratrol-induced PD-L1 upregulation. Mechanistically, resveratrol-activated SirT1 promotes Snail protein stability through its deacetylation. Snail in turn inhibited transcription of Axin2, which leads to disassembly of destruction complex and enhanced binding of β-catenin/TCF to PD-L1 promoter. Our work herein implies the role of resveratrol in tumor immune evasion.

Resveratrol was widely known as a chemopreventive agent against tumor transformation, recent studies have also indicated that, besides chemopreventive effects, resveratrol also exhibits anti-cancer therapeutic properties (Aluyen et al. 2012). Its potential as a therapeutic agent is due to the regulation of many signaling pathways critical in tumor cell proliferation, apoptosis and metastasis. Moreover, some data suggested that resveratrol could sensitize tumor cells to chemotherapeutic drugs and exert additive inhibitory effect with cisplatin (Hsieh and Wu 2010). Prompted by above findings, resveratrol has been suggested as adjuvant to be used in combination with existing primary therapeutic agents. However, it has been reported that the anti-cancer activities of resveratrol were achieved at relatively high, non-physiological concentrations (usually > 50 μM), and at relatively lower concentrations resveratrol showed growth-stimulatory and metastasis-promoting activities (Castillo-Pichardo et al. 2013; Fukui et al. 2010a, b). Consistently with the effect of resveratrol appearing to be dose- and cell-type dependent, we found that regulation of PD-L1 expression by resveratrol is also in a dose-dependent manner and suppression of T cell function at relatively low concentrations of resveratrol. Given that resveratrol is widely used as dietary supplement among cancer patients, our study is relevant to the debate on the potential benefits and perils of resveratrol as an adjuvant in cancer therapy.

Resveratrol has shown a unique ability to modulate multiple signaling pathways including Wnt, TGFβ1, Notch and STAT in various types of cancer (Farooqi et al. 2018). In this work, we have presented evidence that resveratrol activates Wnt pathway to upregulate PD-L1 expression. Our finding about molecular mechanism of resveratrol-induced PD-L1 expression is not in line with some previous studies showing that resveratrol suppressed Wnt signaling and inhibited the nuclear translocation of β-catenin (Chen et al. 2012; Cilibrasi et al. 2017). However, in those studies, resveratrol seems to target other molecular targets rather than directly targeting the components of destruction complex of Wnt pathway. Moreover, there are also reports that resveratrol is capable of activating Wnt/ β-catenin pathway in a dose-dependent manner (Wang et al. 2010; Zhang et al. 2009). Thus, our data reinforces the notion that control of Wnt pathway by resveratrol is probably regulated in a distinct manner in different types of cancer and is also concentration-dependent. The finding that resveratrol upregulates PD-L1 expression by activating Wnt pathway is consistent with recent reports that dysfunction of Wnt pathway significantly altered PD-L1 expression in triple-negative breast cancer and suppression of Wnt signaling by IL-21 reduced PD-L1 expression in NSCLC cells (Castagnoli et al. 2019; Xue et al. 2019).

We find that Axin2 is reduced by Snail and that this effect is driven by resveratrol-activated SirT1. This result confirms indirectly the report that Snail can functions as transcriptional activator or repressor and acetylated Snail generally binds the promoters of activated targeted genes, while deacetylated Snail binds to the promoters of repressed targeted genes. Our study also provides complementary evidence that both Snail levels and function can be regulated by acetylation. Importantly, we first time demonstrated that Snail can control Wnt pathway through reduction of destruction complex component Axin2 to increase PD-L1 expression. This finding was corroborated by evaluation of the potential correlations among Snail, Axin2 and PD-L1 using publicly available data sets from TCGA and GEO.

Given the fact that many studies have suggested that resveratrol have a number of health beneficial effects and many cancer patients are taking resveratrol as dietary supplements, our results implicate that physiologically relevant concentrations of resveratrol has potential to promote of tumor immune evasion—a finding that warrants further exploration.

## Data Availability

The data used and analyzed in this study are available from the corresponding author on reasonable request.
